# Using Facebook to reduce smoking among Australian Aboriginal and Torres Strait Islander people: a participatory grounded action study

**DOI:** 10.1186/s12889-019-6918-7

**Published:** 2019-05-21

**Authors:** Marita Hefler, Vicki Kerrigan, Becky Freeman, Gordon Robert Boot, David P. Thomas

**Affiliations:** 1 Tobacco Control Research Program, Wellbeing & Preventable Chronic Diseases Division, Menzies School of Health Research, PO Box 41096, Casuarina, NT 0811 Australia; 2 Prevention Research Collaboration, School of Public Health, Charles Perkins Centre, Sydney Medical School, The University of Sydney, Sydney, NSW 2006 Australia; 3 Miwatj Health Aboriginal Corporation, PO Box 519, Nhulunbuy, NT 0881 Australia

**Keywords:** Smoking, Social media, Health communication, Qualitative research, Indigenous health

## Abstract

**Background:**

There is limited evidence for the effectiveness of social media to promote healthy behaviour among Indigenous Australians, including to reduce smoking. Social media has significant potential to stimulate interpersonal influence to quit, however an important knowledge gap is how and what content people choose to share with friends and family. This paper explores the decision making processes of community members for sharing tobacco control content with family and friends on Facebook.

**Methods:**

Community researchers were paid to choose and share at least one tobacco control post per week for a period of 6 months on their personal Facebook page. They documented reasons for their choices, which were coded and analysed to determine features of messages most likely to be shared, and salient considerations in the decision-making process.

**Results:**

Posts which are child-focused, feature Indigenous content, and are perceived as practical, relevant and credible, with a direct and unambiguous message, were most likely to be shared. Posts which included disgusting imagery about health impacts, were focused on the environment, or were ambiguous or sarcastic were less likely to be shared. Decisions were also based on whether content was perceived to contain new information, to be helpful for their friends, and to be consistent with the participant’s online identity, as well as the perceived sensitivity of content. The potential impact on expensive mobile data for videos was also a factor.

**Conclusions:**

When designing tobacco control messages to be shared on social media, health promoters should take into account how information will align with positive self-image and can contribute to social capital among the intended audience, and generate interpersonal engagement. Content should complement, rather than attempt to replicate, some message features that are effective on traditional broadcast media. This study shows the potential for health services to incorporate a strategy of using paid local social media ‘champions’ or ‘ambassadors’ to disseminate tobacco control messages on Facebook through community networks.

## Background

For Australian Aboriginal and Torres Strait Islander peoples, social media have the potential to encourage social support and enact an agenda of self-determination and empowerment which aligns with Indigenous notions of health [1, 2]. Although limited current statistics are available, research suggests social media use is higher among Aboriginal and Torres Strait Islander people than non-Indigenous Australians [3]. A 2014 survey found that 60% of Indigenous people used Facebook, compared to 42% of the Australian population at that time [4], and in most communities Facebook use has continued to increase since then. Aboriginal and Torres Strait Islander organisations and leaders have been at the forefront of social media use to advance health [5], particularly for online activism, community development, advocacy, citizen journalism, and countering racism and negative stereotypes [2, 5–9]. Social media are used by Aboriginal and Torres Islander people in a range of ways which can impact health and wellbeing, including developing and expressing Indigenous identity [10–12], help seeking and support for suicide and self-harm [13], and communication and support connected to death and grieving [14]. However, there is limited evidence for the effectiveness of social media to promote healthy behaviour among Indigenous Australians, [15, 16] including to reduce smoking [17].

Although the interactive nature of social media is the key to their potential power to achieve health promotion goals [18–20], many health organisations are failing to harness these capabilities [21]. Research about health promotion using social media tends to focus on approaches that use social media as a one-way tool for health education [1]. How to maximise engagement is a challenge not unique to health promotion; research focused on generating consumer engagement for commercial purposes has highlighted a similar tendency towards broadcasting content that involves passive consumption [22]. Both health and commercially-focused research highlights the importance of a user-centred approach to engaging target audiences, taking into account the interpersonal aspects of social media use, its role in building social capital and relationships, and interaction with consumers’ self-image and identity [22–24].

There is an emerging body of research about characteristics of highly shared health content on social media [25–27], however there is little existing research which examines decision-making processes for sharing health-related content with friends and family on social media. Given the potential for peer-to-peer influence through social media [28, 29] and the potential to use social media networks to shape health behaviours [30], this is an important knowledge gap [20]. This is particularly so for tobacco control, given that anti-smoking messages can stimulate interpersonal influence to quit [31, 32].

Tobacco use is a leading cause of disease and premature mortality, and reducing smoking prevalence among Aboriginal and Torres Strait Islander people is an important Australian health priority. Smoking prevalence is around three times higher among Indigenous than non-Indigenous Australians (39 and 14% respectively, in 2014–5 for people aged 15 years and over), however there is limited Indigenous-specific evidence for most tobacco control approaches [17]. Internationally, there is a strong evidence base for mass media communication as part of a comprehensive tobacco control approach, with effectiveness evident across different population groups [33–35]. Messages about negative health effects are most effective at generating increased knowledge and promoting quitting, however impact depends on sufficient intensity and duration of exposure [33]. Achieving exposure at the level necessary to have an impact may be unlikely through social media, as users can control how and to what extent they engage with content, and exposure is highly dependent on sharing from personal contacts. There is therefore a need to understand what tobacco control messages are most likely to be shared through social media, and how messages may need to be adapted to maximise impact.

This study forms part of a larger project to investigate how social media can be effectively used to enhance Indigenous tobacco control in Australia. The first exploratory study for the project found that tobacco control content was rarely shared or seen by participants on Facebook, the most commonly used social media platform [36]. In this study we nested smoking prevention messages within existing Facebook networks, by employing community-based peer researchers (hereafter called participants) to share tobacco control content on their personal Facebook pages. The aim of this paper is to explore the decision-making processes of what content was shared.

## Methods

A ‘grounded action’ [37] approach based on a combination of grounded theory [38] and participatory action research [39] was used. It combined a structured intervention with qualitative data about decision-making processes for sharing content. The study duration was 26 weeks, from December 2016 to June 2017. Consultation with participants about the study design occurred from September to December 2016, in order to ensure maximum participation and minimal burden.

### Participants, recruitment and sampling

Thirteen participants were employed on a casual basis at study commencement. Inclusion criteria were: (1) the participant identified as Aboriginal and/or Torres Strait Islander, (2) was a regular Facebook user (at least weekly), and (3) a significant proportion of their Facebook network was people with whom they had an existing offline connection and regular contact. Participants were recruited from Darwin and Alice Springs, Northern Territory, Australia. Eleven participants had been employed in the initial exploratory study of the project which investigated what types of health-related content is being shared by Indigenous people on social media [36]. Two additional participants were recruited for this study – one recommended by a participant in the previous study, and one responded to information distributed through local community networks. We sought to achieve maximum variation in terms of gender, age, residential location, socioeconomic and employment/study status, and smoking status. Two participants were male, all others were female. The age range was from early 20s through to approximately 60. One participant was a never- smoker, three were ex-smokers. All others were current smokers at study commencement. Four identified as not currently ready to quit, while all others were either contemplating or actively trying to quit.

### Study intervention

Participants were asked to share at least one tobacco control post per week for the study duration. Three options were provided each week, drawn from a content library created by the research team (MH and VK). It contained video and still images, international content, Australian content (general, as well as tailored for Aboriginal and Torres Strait Islander people), content produced for government campaigns, and content created by non-government organisations and others. Selected content was not screened for evidence of impacting smoking attitudes and behaviour, however it was assessed to ensure it did not inadvertently promote smoking, contain factually incorrect information, or was produced by the tobacco industry or entities working to promote its interests. The full list of content options for each week is provided as Table 1. Participants were free to post more than one option each week, not post anything if they did not feel any options were suitable, or to post alternative content they had sourced or created themselves (subject to screening by the project team).

**Table 1:** Weekly content options provided to peer researchers

Week	Option A	Option B	Option C
1.	Video: Catmageddon (Truth Initiative, USA) https://www.youtube.com/watch?v=tLtschJxRy8	Video: 1200 die (American Legacy Foundation) https://www.youtube.com/watch?v=4XmyGQJ2WWE	Image: Life meter Description: shows a cigarette with numbers to represent lifespan, with the ash burning towards the number 60. URL not provided as original source not able to be determined.
2.	Video: Quit for you, quit for two (Australian Government campaign, pregnancy focus) http://www.quitnow.gov.au/internet/quitnow/publishing.nsf/Content/quit-you-quit-two	Video: Skinnyfish Yaka Ngarali (created by local record label and Aboriginal community controlled health service) https://www.youtube.com/watch?v=9yPwNVaOPMY&list=PLhVW2l7sc6giYqfvL9qN6l0Q89kx0vAzd&index=1	Image: Smoking burns money Cartoon-like image showing a hand holding a lit cigarette, with the ash replaced by coins. URL not provided as original source of image not able to be determined.
3.	Video: Break the Chain (Australian Government campaign, Indigenous focus) https://www.youtube.com/watch?v=0yvjBU-E0aw	Video: 5 things the tobacco industry doesn’t want you to know (produced by Public Health Ottawa) https://www.youtube.com/watch?v=UB1biuoDkEQ	Image: smokers teeth Description: photo of young attractive women holding a cigarette and smiling, showing extremely strained teeth. URL not provided as original source not able to be determined.
4.	Video: don’t make smokes your story (Australian Government campaign, Indigenous focus) https://www.facebook.com/healthgovau/videos/1089321171111579/?v=1089321171111579	Video: Allen Carr top tips on quitting https://www.youtube.com/watch?v=0TL2Vh7goJc	Image: Smoking good for environment Close up photo of lower half of face with cigarette and caption “Smoking is good for the environment because it kills humans”. URL not provided as original source not able to be determined.
5.	Video: Bryan Curtis story (YouTube user generated content) https://www.youtube.com/watch?v=dVLtNgAhPRg	Video: Quit smoking timeline Video of how the body recovers from smoking. Produced by online quit smoking community www.quitsmoking.com. https://www.youtube.com/watch?v=fLbQfMmrISE	Image: Dream boy Poster with a photo of a young Indigenous boy dressed for boxing, and the caption “Fill my head with dreams, not my lungs with smoke”. Current poster URL not available. Website listed on poster: www.giveupsmokesforgood.org.au
6.	Video: Thai smoking kids Advertisement produced by Ogilvy Asia for the Thai Health Promotion Foundation. https://www.youtube.com/watch?v=qHH2LsAHeHc&feature=youtu.be	Video: Social farting/smoking Available from Middlesex London Health Unit YouTube channel. https://www.youtube.com/watch?v=9rIj2DWQRdw	Image: smokers’ funeral Photo of two people smoking in a room, with the ceiling painted to look like they are looking up from a grave with people attending their funeral looking on. URL not provided as original source not able to be determined.
7.	Video: Everybody knows Video created by Cancer Institute NSW, which shows clips from a range of well-known Australian anti-smoking campaigns, set to Leonard Cohen song “Everybody knows”. Link not provided, as original URL not able to be determined.	Video: Cigarettes & child labour Information video produced by AJplus news media. https://www.youtube.com/watch?v=t6pVsA6hdYs	Image: cup of butts Photo showing a teacup full of cigarette butts and a teaspoon with a butt above it. URL not provided as original source not able to be determined.
8.	Video: Culture or killer Produced for Lakes Entrance Aboriginal Health Association in Victoria, Australia. https://vimeo.com/127003575	Video: smoking and drinking. Video showing animation of the impacts of smoking and drinking on the body. (Appears to be YouTube user-generated content) https://www.youtube.com/watch?v=XXwpTrI74BQ	Image: Quit gains Poster with the question “What have you gained from quitting?” in the centre, surrounded by line drawings to suggest improved health and money saved. URL not provided as original source not able to be determined.
9.	Video: exercise & quitting https://www.youtube.com/watch?v=-dHzc4xqHks	Video: smoking damage. Motion graphic video on health impacts of smoking. Australian government, Indigenous focus. https://www.youtube.com/watch?v=IfIBckA0hAc	Image: smoking increases stress Monotone mage of a cigarette pack shaped like a coffin, with the words “Smoking reduces stress. *False*” in red. Further down “Although smoking temporarily relieves the stress of withdrawal symptoms, scientists have found that nicotine actually increases your level of stress hormones” is printed in black. URL not provided as original source not able to be determined.
10.	Image: Quit steps Australian poster with 7 steps, accompanied by graphics, to help with quitting smoking: set a date, banish ashtrays, call the Quitline (with Quitline number prominently included, exercise, snack on healthy foods, drink water and have drinks with less alcohol. URL not provided as original source not able to be determined.	Image: start repairing Australian government poster with a photo of a person, pointing to different parts of the body, and examples of both short and longer-term (5 days up to 1 year) benefits of stopping smoking.	Video: Real cost – your skin US Government Food & Drug Administration campaign advertisement. Can be viewed at https://www.ispot.tv/ad/7BnU/the-real-cost-your-skin
11.	Image: pet smoking Form the USA Truth campaign, image of the words “Fact: dogs and cats are twice as likely to get cancer if their owner smokes”.	Video: Donna, Alice Springs Don’t make smokes your story. Australian Government campaign, Indigenous focus, with real local people. https://www.youtube.com/watch?v=rOy1vO-HlnA	Image: Trigger Poster with the heading “if you smoke…” With four boxes containing animated graphics and the words: “In the morning, try a new morning routine”, “after meals, have a mint or a cup of tea instead”, in your car, clean it out to get rid of the smell and keep healthy snacks with you” and “when you’re stressed, get enough sleep, stay active and talk about what is bothering you”. Produced by the Massachusetts Tobacco Cessation and Prevention Program, USA. URL of poster not provided as original source not able to be determined. Department website: https://www.mass.gov/massachusetts-tobacco-cessation-and-prevention-program-mtcp
12.	Image: appearance change. Shows photo of a woman with one side as a non-smoker aged 35 and the other side as a smoker of the same age, highlighting the differences and appearance impacts of smoking. Queensland Health, Australia campaign. Image can be viewed at https://www.couriermail.com.au/news/queensland/smoking-makes-you-ugly-queensland-health-to-tell-young-women/news-story/d80e3e017b01d636d692ed884113ef41	Image: Quit tip Image from Australian Government campaign Don’t Make Smokes Your Story, with the words “#QuitTip Ask friends who smoke to promise not to offer you any cigarettes”. Current poster URL not available. Campaign website: https://web.archive.org/web/20190304210745, https://campaigns.health.gov.au/smokes	Image: smoking sarcasm Cartoon like image of a lit cigarette with arms ‘eating’ a person through the lit end of the cigarette. Accompanied by a caption “Share if you are against smoking”. URL not provided as original source not able to be determined.
13.	Image: Quit tip morning Image from Australian Government campaign Don’t Make Smokes Your Story, with the words “#QuitTip Take it 1 day at a time. Every morning say, “I’m not going to smoke today””. Current poster URL not available. Campaign website: https://campaigns.health.gov.au/smokes	Image: timebomb Photo of a bundle of cigarettes tide to a clock to resemble a time bomb, with a hand with a lighter about to light the fuse. Accompanied by the caption “Every breath you take will eventually destroy your future”. URL not provided as original source not able to be determined.	Image: Butts are litter Image of a cigarette being stubbed out with the words “Cigarettes butts are LITTER too…” URL not provided as original source not able to be determined.
14.	Image: fish butts Image of internal cross-section of a fish with butts in the stomach cavity, accompanied by a piece of paper which resembles a shopping docket with information about how cigarette butts contaminate water. URL not provided as original source not able to be determined.	Image: Four D’s Image of a poster with the heading “Practice the Four D’s to help you get through a craving. Each D is listed (Delay, Drink water, Deep breathing, Distract) with explanatory text and a graphic. Produced by the Massachusetts Tobacco Cessation and Prevention Program, USA. URL of poster not provided as original source not able to be determined. Department website: https://www.mass.gov/massachusetts-tobacco-cessation-and-prevention-program-mtcp	Video: Sugar sugar Produced for Make Smoking History campaign by Cancer Council WA, Western Australia. Campaign website: https://makesmokinghistory.org.au/ https://www.youtube.com/watch?v=0fXdLCTND1A
15.	Image: Quit fact Image from Australian Government campaign Don’t Make Smokes Your Story, with the words “#QuitFact within a day of quitting, almost all the nicotine is out of your bloodstream”. Current poster URL not available. Campaign website: https://web.archive.org/web/20190304210745, https://campaigns.health.gov.au/smokes	Video: Make smoking history (please help me quit) Produced for the Cancer Council WA Make Smoking History campaign, Western Australia. Campaign website: https://makesmokinghistory.org.au/ https://www.youtube.com/watch?v=80gkwcx314w	Video: lost child Video produced for Cancer Council Victoria, Australia. Information about the ad can be viewed here: https://www.cancervic.org.au/about/media-releases/2008-media-releases/media-rel-october08/quit-launches-separation-08.html Link to video: https://www.bestadsontv.com/ad/17507/Quit-Victoria-Separation
16.	Image: No more killing Image of two burning cigarettes placed upright to resemble the twin towers on fire after the 9/11 attacks. Accompanied with the words “NO MORE Killing” and in small type “It is estimated that one person dies every 8 s from smoking. Stop smoking now!” URL not provided as original source not able to be determined.	Image: Happy ending Poster with a photo of a young Indigenous girl in a T-shirt printed with an Aboriginal flag, with the caption “Stories about smokers never end with happily ever after”. Current poster URL not available. Poster includes the URL https://www.instagram.com/p/Bso_iAvlvc4/	Video: Every cigarette rots you from the inside out NHS England Smoke Free campaign https://www.youtube.com/watch?v=7ctaMwtHwUo
17.	Image: smoking gun Black and white photo of a hand holding a cigarette against a blank wall, with the shadow resembling a pointed gun. URL not provided as original source not able to be determined.	Video: I will survive Video produced for HSE Ireland. Campaign website: www.quit.ie. https://www.youtube.com/watch?v=NSETW_zO9jc&feature=youtu.be&list=PLsQK32cdMW_w6X1OiaNXOjwbD2XKbcS1r	Video: Don’t make smokes your story. Australian Government campaign, Indigenous focus, with real local people. https://www.youtube.com/watch?v=em_0c_Yzxyk
18.	Image: poor swimmers Photo of used matches resembling sperm swimming in an ashtray towards ash (representing an ovum). Produced for ASH UK, image can be viewed at https://www.flickr.com/photos/ashuk/4459586151.	Video: Message from the lungs Video produced for the Thai Health Promotion Foundation. https://vimeo.com/126220314	Image: cancer cures smoking Image of the words “Cancer cures smoking”. No other graphics or pictures are included. Taken from https://www.thefreshquotes.com/50-smoking-and-tobacco-quotes-and-slogans/cancer-cures-smoking/.
19.	Image: baby bottle Photo of a baby’s bottle half-filled with cigarette butts and smoking coming out of the top, accompanied with the words “How many cigarettes a day does your child smoke?” at the top, and “Prevent passive smoking” in smaller letters at the bottom. URL not provided as original source not able to be determined.	Video: Plain packaging Video produced for World Health Organization for World No Tobacco Day 2016, which had the theme “Get ready for plain packaging.” https://www.youtube.com/watch?v=rXUCTSp2_58	Image: pregnant Poster with a photo of a pregnant belly with a lit cigarette held next to it and smoke appearing to come out of the belly button. URL to poster not available, organisation website: www.kindergesundheit.de.
20.	Image: squirrels Photo of a squirrel with the wording “Cigarettes are like squirrels: they’re perfectly harmless until you put one in your mouth and light it on fire.” URL not provided as original source not able to be determined.	Video: Quit smoking (Arrente) Animated video of a brain explaining the impact of smoking in Arrente, a central Australian Aboriginal language. Available at: http://nosmokes.com.au/teachers-and-health-workers/language-translations/central-arrernte/	Image: child labour pack Photo of a an open pack of cigarettes with children inside the pack instead of cigarettes, and in place of the health warning label, the words “Made with child labor”. Image can be viewed at https://www.hrw.org/news/2014/05/14/us-child-workers-danger-tobacco-farms
21.	Video: Tips from former smokers (gum disease) Video produced from the “Tips from Former Smokers” campaign by the USA Centers for Disease Control. https://www.youtube.com/watch?v=y70V5HGhpHs	Image: Weight loss Image of the words “Smoking reduces weight” in large print with “(one lung at a time)” in smaller print below. The image has the logo and name of Cancer Patients Aid Association. URL not provided as original source not able to be determined.	Image: impotent Photo of a partially burned cigarette half ash, with the word “Warning” in red, followed by “Tobacco use can make you impotent” in large letters, and in smaller letters “Cigarettes may cause sexual impotence tdue to decreased blood flow to the penis. This can prevent you from having an erection”. The image was used by Health Canada as a cigarette warning label, 2010–2011. The image can be viewed at https://www.who.int/tobacco/healthwarningsdatabase/tobacco_large_canada_impotence_01_en/en/
22.	Image: Piggy bank Photo of a child’s piggy bank with cigarette butts stuffed into the slot where money is usually placed. Ash and an additional cigarette butt are around the piggy bank. The words “Daddy couldn’t give me pocket money” are below the image in handwritten, coloured crayon style. The image has the logo of the British Heart Foundation in the bottom right hand corner, current poster URL not able to be located.	Image: dirty lungs Photo of two sets of preserved lungs – one white, one partially blackened due to tar. Not caption or accompanying text. URL not provided as original source not able to be determined.	Video: stop smoking, start repairing Australian Government video to promote the My QuitBuddy app. https://www.youtube.com/watch?v=e7iGNrdST6E&feature=youtu.be
23.	Image: smoke mouth Stylised mage of a woman’s open mouth with glossy bright lipstick. The mouth is full of cigarette butts, and there is a cigarette hanging from the mouth with the ash end in the mouth. URL not provided as original source not able to be determined.	Image: smoking kids Portrait style photograph of a young red-haired girl in a blue dress lighting a cigarette from another one. Image available at http://frieke.com/smoking-kids/.	Image: pet 2nd hand smoke Photo of a dog and cat with a sign hanging around their necks with the words “dogs/cats against smoke” and a non-smoking symbol. At the top are the words “Secondhand smoke hurts them too. Keep your whole family safe…keep your car and home smoke-free.” URL not provided as original source not able to be determined.
24.	Video: Make smoking history (Darleen’s story) Produced for the Cancer Council WA Make Smoking History campaign, Western Australia. Campaign website: https://makesmokinghistory.org.au/ https://www.youtube.com/watch?v=r8yvgQFWObA	Image: savings Image from Australian Government campaign Don’t Make Smokes Your Story, with the words “By quitting smoking, a daily smoker could save $8500 a year”. Current poster URL not available. Campaign website: https://web.archive.org/web/20190304210745, https://campaigns.health.gov.au/smokes	Image: not our culture Image of words “Not Our Culture” next to images associated with Aboriginal culture and a cigarette crossed out. Image can be viewed at https://nacchocommunique.com/tag/ahl/
25.	Image: blindness Photo of a public rubbish bin with top designed to resemble an eye and a cigarette being stubbed out on the iris. Around the bottom of the eye white are the words “Smoking causes blindness” with a Quit logo. URL not provided as original source not able to be determined.	Image: baby lungs Poster with a photo of a baby in a light coloured jumpsuit, with blackened out lungs on the jumpsuit. At the bottom are the words “You smoke, your child smokes! Take your smoke outside. By the time he is 6 years old your child will have inhaled the equivalent of 102 packs of cigarettes”. Produced for Tobacco Free Futures (http://www.tobaccofreefutures.org/), current URL for poster not located.	Video: Tobacco, a threat to development Video produced for World Health Organization for World No Tobacco Day 2016, which had the theme “Tobacco: a threat to development.” https://www.youtube.com/watch?v=B9xqy6g8SIw
26.	Video: Miwatj – cost of smoking Video created by Aboriginal community controlled health service for World No Tobacco Day 2017. https://vimeo.com/220741839	Video: Apunipima – Selena’s story Video produced by a local Aboriginal community controlled health service https://www.youtube.com/watch?v=LMPcoA6cz_Q	Image: diabetes Poster with graphic of cigarette on brown background on left side, and finger prick blood test on red background on right side. In the middle, inside a circle made up of 2 arrows are the words “Smokers have a 30 to 40% higher risk of diabetes than nonsmokers”. The poster has a US Centers for Disease Control and one other logo, and has appeared in campaigns and on social media for several US health groups. (Eg see https://twitter.com/tobaccofreekids/status/978745983923142657 and http://www.coprevent.org/2014/11/national-diabetes-month-smoking-and.html). URL not provided as original link not able to be determined.

### Data collection

Participants were asked to: document the reasons for the content they did and did not select, and track the interactions with each post – both online (e.g. likes, comments, shares), and offline (e.g. discussions prompted by post content). This information, together with screenshot(s) of the content posted was sent to the research team each week. The research team interviewed the participants each month to clarify or collect additional information about the decision-making process for selecting posts. Participant quotes in the results section are taken from both interviews and written information sent by text or email.

### Data analysis

Data was inductively coded, adapted from a grounded theory approach, using Microsoft Word to create codes and record memos [38]. Initial coding was developed by the research team and was based on content characteristics, using both screenshots of the Facebook posts and the information about decision-making provided by participants each week. Additional data collected through monthly interviews was also coded, and insights added to memos. An iterative approach was used; as categories were developed, these were compared to earlier data from each participant, and interpretations were checked with participants to allow their feedback to be integrated. A final reflection interview was held with each participant to further refine and develop the categories developed through the 6 months of data collection. At the completion of the data collection period, all posts were sorted by popularity based on the total number of times they were posted by participants, (provided as Table 2) and the percentage of participants each week who shared the post, as well as broad themes of the content. The quantitative and qualitative analyses were then compared to further develop the key themes. The initial collated findings were presented to the participants and project partners at a project meeting held in September 2017, for further development, refinement and validation. In particular, discrepancies regarding posts that appeared to be popular based on quantitative analysis and participants’ perceptions reported in interviews were discussed and resolved. The themes presented here represent consensus agreement about themes that shaped participants’ choices.

**Table 2:** Content options by popularity

No. of participants for week	Post ID (week/choice)	Title	No. of participants who posted this content option	% of participants who posted this content option	Total number of posts by participants for the week^a^	Content characteristics/themes
10	WEEK 12A	Appearance change: IMAGE	9	90	14	Personal impact, visible damage
10	Week 6A	Thai Smoking Kids: VIDEO	8	80	12	Kid focused, unique/fresh take on message, role modelling
9	WEEK 13A	Quit Tip Morning: IMAGE	8	89	13	Practical, quit tip
11	Week 4A	Don’t Make Smokes yr story: VIDEO	7	64	12	Testimonial, Aboriginal, family/kids focus, positive
8	Week 21C	Impotent: IMAGE	7	88	15	Humorous approach, sexual health, male focus
8	Week 26A	Miwatj money: VIDEO	7	88	14	Personal, Local NT Aboriginal (Yolngu) content, finance
12	Week 5B	Quit Timeline: VIDEO	6	50	14	Positive, benefits of quitting
9	Week 7B	Child Labour Tobacco: VIDEO	6	67	13	Child-focused, new information, targets industry
8	WEEK 11B	Alice Springs: VIDEO	6	75	12	Testimonial, Local NT Aboriginal content, family/kids focus, positive
9	WEEK 15A	Quit Fact: IMAGE	6	67	13	Positive benefits/fact re quitting
8	WEEK 16B	Happy Ending: IMAGE	6	75	12	Aboriginal content, child focused
9	Week 22C	Quit Buddy App: VIDEO	6	67	15	Practical, quit tip, based on personal experience
8	Week 24B	Savings: IMAGE	6	75	14	Financial focus
9	Week 25A	Blindness: IMAGE	6	67	14	Health impact (new/lesser known)
12	Week 5C	Dream boy: IMAGE	5	42	14	Aboriginal content, child focused
8	Week 8B	Culture or Killer: VIDEO	5	63	10	Aboriginal content, culture focus
7	Week 10A	Quit Steps: IMAGE	5	71	11	Practical quit tips
8	WEEK 11C	Trigger: IMAGE	5	63	12	Practical quit tips
8	WEEK 14B	Four D’s: IMAGE	5	63	11	Practical quit tips
9	WEEK 15C	Lost Child: VIDEO	5	56	13	Child-focused, family
8	Week 18A	Poor Swimmers: IMAGE	5	63	9	Humorous/fresh approach, sexual health, male focus
8	Week 20C	Child labour pack: IMAGE	5	63	11	Child-focused, new information, targets industry
8	Week 21B	Weightloss: IMAGE	5	63	15	Negative, health impacts, non-obvious message
9	Week 22A	Piggy Bank: IMAGE	5	56	15	Financial focus
8	Week 23A	Smoke mouth: IMAGE	5	63	11	Negative, personal (cosmetic) impact
9	Week 25B	Baby lungs: IMAGE	5	56	14	Child-focused, protection from smoke impact
8	Week 26B	Selena Story: VIDEO	5	63	14	Testimonial, Aboriginal content, family/kids focus, Top End
8	Week 1C	Lifemeter: IMAGE	4	50	8	Negative, health impacts
11	Week 3B	5 Things, Tobacco Industry: VIDEO	4	36	11	Negative, health industry focus, uninteresting thumbnail
11	Week 3C	Smokers teeth: IMAGE	4	36	11	Negative, appearance focused
9	Week 7A	Everybody Knows, Quitline: VIDEO	4	44	13	Negative, health impacts
8	Week 9B	Damage Lesson: VIDEO	4	50	11	Negative, health impacts
7	Week 10B	Start Repairing: IMAGE	4	57	11	Health impacts
8	Week 14A	Fish Butts: IMAGE	4	50	11	Environmental focus, negative
8	Week 16A	No More Killing: IMAGE	4	50	12	Indirect/non-obvious message
9	Week 17B	I Will Survive: VIDEO	4	44	11	Negative, health impacts
9	Week 17C	Kirra Story: VIDEO	4	44	11	Local content, testimonial, child focused
9	Week 19A	Baby bottle: IMAGE	4	44	11	Negative, indirect
9	Week 19C	Pregnant: IMAGE	4	44	11	Pregnancy focus
8	Week 20B	Arrernte: VIDEO	4	50	11	Local content
9	Week 22B	Dirty Lungs: IMAGE	4	44	15	Gross, biomedical focus
8	Week 23C	Pet 2nd hand smoke: IMAGE	4	50	11	Pet focused
8	Week 24A	Personal story: VIDEO	4	50	14	Local content, testimonial
8	Week 24C	Not our culture: IMAGE	4	50	14	Aboriginal, culture focus
8	Week 1A	#Catmaggedon: VIDEO	3	38	8	Pet focused, humorous
8	Week 2B	Bolypingu, Skinnyfish: VIDEO	3	38	8	Local Aborigjnal content, indirect message
8	Week 2C	Smoking burns money: IMAGE	3	38	8	Financial focus, cartoon
11	Week 3A	Break the Chain: VIDEO	3	27	11	Aboriginal focus, testimonial
11	Week 4B	Quit Smoking, Allen Carr:VIDEO	3	27	12	How to quit focus
12	Week 5A	Smoking Kills, Bryan Curtis: VIDEO	3	25	14	High negative emotion
10	Week 6C	Smokers Funeral: IMAGE	3	30	12	Negative, unclear message
9	Week 7C	Cup of butts: IMAGE	3	33	13	Negative, focus on taste/smell
8	Week 8C	Quit gains: IMAGE	3	38	10	Indigenous content, unclear message
8	Week 9A	Exercise: VIDEO	3	38	11	Practical tips
8	Week 9C	Stress: IMAGE	3	38	11	Indirect, stress focus, unclear message
10	WEEK 12B	Quit Tip: IMAGE	3	30	14	Practical tips
9	WEEK 13B	Timebomb: IMAGE	3	33	13	Negative focus
9	WEEK 17A	Smoking Gun: IMAGE	3	33	11	Negative, unclear/indirect message
8	Week 18C	Cancer cures smoking: IMAGE	3	38	9	Sarcasm, negative
9	Week 19B	Plain packaging: VIDEO	3	33	11	Official, tobacco packaging focus
8	Week 20A	Squirrels: IMAGE	3	38	11	‘Silly’ humour
8	Week 21A	Gum Disease: VIDEO	3	38	15	Negative, health impacts, gross
9	Week 25C	World No Tobacco Day: VIDEO	3	33	14	Boring, no new information
8	Week 2A	Quit for you, Quit for Two: VIDEO	2	25	8	Pregnancy focus
11	Week 4C	Smoking good for environment: IMAGE	2	18	12	Environment focus, sarcastic, insensitive, cruel
8	Week 8A	Smoking and drinking: VIDEO	2	25	10	Health impacts, biomedical
7	Week 10C	Real Cost - Your skin: VIDEO	2	29	11	Cosmetic impacts, gross
10	Week 12C	Smoking sarcasm: IMAGE	2	20	14	Cartoonish, potentially hypocritical
9	Week 13C	Butts are Litter: IMAGE	2	22	13	Environmental focus, message not important
8	Week 14C	Sugar Sugar: VIDEO	2	25	11	health damage, sad and tragic
9	Week 15B	Smoking History: VIDEO	2	22	13	Clashes with idea of respect for others, quitting own responsibility
8	Week 16C	Rotting: VIDEO	2	25	12	health impacts, gross
8	Week 23B	Smoking kids: IMAGE	2	25	11	Indirect, Image looked like it was promoting kids smoking
8	Week 26C	Diabetes: IMAGE	2	25	14	Health impacts
8	Week 1B	1200 people die: VIDEO	1	13	8	Obscure message, thumbnail misleading & confronting
10	Week 6B	Social Farting/Smoking: VIDEO	1	10	12	Humorous, impolite, crass
8	Week 11A	Pet Smoking: IMAGE	1	13	12	Pet focused
8	Week 18B	Black Ink: VIDEO	1	13	9	Obscure message, thumbnail not appealing

^a^The total number of posts exceeds the number of participants in some weeks. This is due to some participants choosing to post more than one content option for that week

### Ethical issues

Two non-Indigenous researchers (MH & VK) worked in close collaboration with the partner Aboriginal Community Controlled Health Services, and the participants, to ensure an inclusive, respectful and culturally appropriate approach which adheres to Australia’s guidelines for ethical conduct in Aboriginal & Torres Strait Islander health research [40]. Written consent was obtained from participants, who were free to withdraw from the study at any time. Identifying information such as names and profile pictures (of participants and their Facebook friends) was removed from all data during analysis. Pseudonym codes have been used for direct quotes in this article. As several participants were smokers who were in the process of attempting to quit, there was significant potential for them to experience distress. Counselling and other support services were available as needed; several referrals were provided to both general counselling services and Quitline.

## Results

The average number of participants for each week of the study was 8.6 (range 7–12). One community researcher withdrew from the study at 5 weeks, and one posted only one once; all others continued for the duration of the study, with some weeks of non-participation decided by individual participants according to their personal circumstances.

### Content

Seventy eight content options were offered: three per week for 26 weeks. Each option was offered only once.

In the first 3 weeks of the study, the total number of posts matched the number of participants (for example eight participants each posted once for a total of eight posts). Thereafter, in every week, the total number of posts exceeded the number of participants (i.e. some participants posted more than one option). No participant created their own content, and only one posted content (once) not supplied by the research team. All other posts were taken from the content choices provided.

### Characteristics of highly-posted content

Overall, the most popular posts were child-focused, closely followed by Aboriginal-focused posts, with these two themes frequently overlapping. Practical information in the form of quit tips was also a popular choice. Table 2 shows posts by popularity.

#### Child-focused messages

Child-focused messages included: the importance of children and family as a motivator for quitting, the need to protect children from tobacco smoke, and tobacco industry exploitation of child labour. Discussions with peer researchers throughout the project highlighted that a focus on children is a non-threatening way to communicate messages - even smokers who don’t want to quit can agree on the message of valuing and protecting children.

While an Aboriginal and/or local focus was valued by participants and made it more likely that a post would be shared, it was not a necessary pre-condition for sharing child-focused messages. The most shared post was a Thai Health Promotion ad with subtitles (week 6, option A). It shows footage of young children asking for a light from adult smokers, all of whom refuse and cite a range of reasons why the children should not smoke. The children then hand a note to the adults, reminding them that these reasons apply equally to themselves. It was seen to have universal relevance, evoked strong emotions, and highlighted for many people the importance of both role modelling and being consistent in their actions and advice to young people about smoking. This post was shared by eight of the 10 participants who posted that week.

“I have always told my sister’s children not to smoke...it was a good case of do as I say not as I do...my coughing around them was, I thought, testament to that and I thought my li’l gammon* admonishments were enough. No good ...two out of the five children smoke...I did what I could at the time. Three outta five ain’t bad. But at the time of turning 18...it’s an adult choice. But if I found my 14 year old niece smoking...I think I would cry. My niece always used to look at me quizzically as to me telling her not to smoke and puffing away same time. This time I want to prove to her I’m important too...and I shouldn’t smoke either.” (D6, discussing week 6 option A)

(*Gammon is used in the Australian Indigenous context meaning to joke or make a token gesture)

The impact of having children in messages was perhaps best encapsulated by a former smoker:

“I think using children and emotions is the best thing to tackle that because when I talk to a lot of my friends about why they gave up smoking, and why I gave up smoking, the reason is because of our children.” (D4)

While the participants all reported valuing and feeling comfortable sharing messages about children, there was some child-focused content that was poorly received and not selected for sharing. Among the less popular content featuring children were advertisements in which the anti-smoking or ‘protect children’ message was not explicit. One post option in particular - a parody of a tobacco advertisement featuring a child imitating adults by smoking (week 23 option B)– was highlighted by a participant as looking like it “was designed to promote smoking to children”. (D7)

#### Aboriginal and local content

An Aboriginal focus, or content produced in the Northern Territory, was seen as more relatable and relevant to people’s lives than other content. However, an Indigenous or Northern Territory focus was itself not sufficient – if the content was not perceived to be high quality, credible or relevant, it was less likely to be shared. Several messages which had an Indigenous focus were less popular for these reasons. Credibility was particularly important – for example, one Aboriginal-specific option was rejected by several participants who were aware that the woman featured was an actress rather than a person discussing her real life, particularly if they believed she was a never-smoker.

“The other image with the woman telling ‘ her story’. I know the actor and she has never smoked so that illusion didn’t work for me.” (D6)

If people had a personal connection to the person featured (for example, it featured a person known in their friendship or family circles), the importance of supporting local community, and encouraging others was an important motivator for posting - sometimes above the smoke-free message. However, personal connections alone were not sufficient –the content still needed to be perceived as relevant to the lives of both the community researchers themselves and people within their networks.

Content featuring an Indigenous person, telling a story that was relatable and perceived to be genuine, were popular regardless of a local connection, as demonstrated by D1, discussing the reasons for posting content from another state:

“The reason for me to choose this option was to connect followers from [my home area]. Her story people I know could relate to, non-smoker being surrounded by people who are smoking, ended up smoking because of people around her.” (D1, discussing week 26, option C)

In this case, the personal connection of the message to D1’s own family was an important motivator for posting.

#### Practical, useful information

Quit tips were well regarded because participants reported that people know the health impacts of smoking and more attention needs to be paid to helping in a practical way – perhaps reflective of the fact that many participants were smokers who wanted to quit. Information that people could use to change behaviour was highly valued, particularly if it had been personally helpful and was therefore considered credible. It was less important that quit tips contained new information than for other types of content. If community researchers were already aware of a tip, it was considered a good reminder/reinforcement for their own behaviour, and also helpful to share with others.

“As a smoker, I personally experience and became aware of the triggers for me. Triggers seem to be habits for me. A few triggers for me is, jumping in the car to drive and after a meal or having a coffee. I would normally light up a cigarette after these activities… I have cut back dramatically; in turn I don’t drink as much tea and coffee. I have inadvertently cut back on tea and coffee because they go hand in hand with my smoking. I think people would benefit from little reminders like this image to help break the habit.” (D7, discussing week 11, option 7)

### Characteristics of least popular content

The least popular content featured sarcastic, indirect or obscure messages. Content considered likely to have the potential for shame, embarrassment or disgust was also shared less frequently, as was content which placed less importance on people such as the environmental impact of tobacco, or the effect of second-hand smoke on pets.

#### Indirect, obscure and sarcastic messages

Messages which had an unexpected twist in the message were understood in different ways by different people, and frequently misinterpreted. For example, week 21 option B ‘Smoking reduces weight’ (with ‘one lung at a time’ in small print) was understood by several people as simply being a straightforward message that smoking can be helpful for keeping weight off. Similarly, week 20 option A – likening cigarettes to squirrels because both are only dangerous if you put them in your mouth and set them on fire – was seen as silly, but without a clear anti-smoking message.

#### Health impacts, gross imagery

The posts that were least shared about health impacts tended to have a biomedical focus, were not considered engaging or did not present new information. ‘Gross’ content (posts which used imagery participants considered disgusting to communicate the harms of smoking) generated very negative responses which turned people off posting. A clear example was week 16 option C, a video showing that ‘every cigarette rots you from the inside out’ which was seen as disgusting, and without a strong motivating element or new information. Similarly, content about how smoking affects lungs and general ‘smoking kills’ or images of sick people were less likely to be shared.

#### Environmental focus

Content which focused on the environmental impact of cigarettes and tobacco were typically not popular, largely being seen as a secondary issue to human health. A direct, but sarcastic, message that ‘smoking is good for the environment’ (because it kills people) (Week 4 Option C) was rejected because of both the sarcasm, and the fact that it appeared to value the environment over people.

“I felt that this image wasn’t aiming at the effects of smoking with our bodies but aimed at the environment…I didn’t think it would be effective enough for my targeted audience, unless we were more worried about our surroundings more than our health for our bodies…My focus though is us humans, the effects of cigarette smoking on the body.”(A1)

Similarly, one participant chose week 14 option A showing a fish full of cigarette butts. However, the reason was that the participant enjoys eating fish as part of a healthy diet:

“I love cooking fish and I’d be angry if I would find my fish full of cigarettes” (D1)

### Decision-making

Apart from the content itself, several other factors and processes influenced decision-making. It should be noted that in some cases the reasons for why a post *was* chosen were clearer than why some posts *weren’t* chosen. In some weeks, people indicated that they would have been happy to share all options, but did not want to post multiple times in 1 week to avoid tobacco-related posts dominating their feed. In other weeks, the posts overall had little appeal, and it was a case of choosing the ‘least-worst’ option. In contrast, some participants avoided posting content that had the most impact on them personally, particularly if they were smokers:

“Just watched the video and wow that scared me! I wouldn’t have shared it. It does have a strong message but me as a smoker trying to quit its conflicting to share…I know that the consequences and reality of this are real but so real it scares me...maybe if I wasn’t a smoker I would share it to encourage people to give up.” (A3, current smoker trying to quit)

Throughout the study this participant reported being strongly impacted by some content in relation to their own quit journey, but separated this from what they considered appropriate to post to influence others. It was important to this participant to keep their own quit journey private.

### New/different information, well presented

Information that was considered to be new was particularly important for posts about health impacts of smoking; for example the connection between blindness and smoking (week 25, option A). Posts about the impact on sexual health (for example, week 21 option C – impotence, and week 18 option A – poor swimmers), were also chosen for this reason. Conversely, some content about smoking in pregnancy was considered to be repeating a message that participants believed already receives disproportionate attention, and was perceived as potentially adding more stress at a time that can already be difficult for some women. Many participants had close personal experience of smoking during pregnancy, and appreciated the complexity of the lives of women who may find it difficult to quit at that time.

Presentation was particularly important; some posts were considered to have good information and content, but were not chosen because they were presented in a way that was boring, not relevant to peoples’ lives, or the message was too long (for videos). The thumbnails and titles for videos were also very important - if they were not clearly related to the topic or sparked interest, they were usually not selected. Participants noted they would not have watched some videos if they were not involved in the project – for example, the ‘1200 people die’ video (week 1 option B), which depicts the fact that 1200 people die from smoking each day in the USA, in a portrayal reminiscent of a mass terrorist event. Several participants remarked they would not have realised it was related to smoking from the thumbnail and title.

### Credibility, personal relevance and experience

Messages that had personal relevance and credibility were seen as authentic and were highly valued by participants, both when deciding what to share, as well as when discussing the personal impact of the content on themselves. This was true for participants at different stages of their quit journeys.

“I believe getting real life stories from smokers telling their journey to quitting is inspirational and works. Real people, real stories, real victories.” (A1, trying to quit and interested in motivating others.)

“[Good to] post onto my timeline with Facebook because telling yourself I’m not going to smoke today is a great motivational seed to plant so you think twice before smoking during the day.” (A5, reflecting on what had been personally helpful to quit and might be useful to others].

Conversely, for some participants, the images that had the most personal impact were less likely to be shared. During a reflection interview at the conclusion of the study, one participant discussed the post that had the most dramatic personal impact, and was the first image recalled when asked about content options offered. The image strongly reminded this person of the loss of a loved one; for this reason they had deliberately not posted it, despite its resonance and the fact that it had helped prompt the person to try to quit smoking.

### Alignment of messages with existing online identity

There was considerable diversity in the extent to which people considered their existing online identity when deciding on appropriate posts. For some participants, their online activity was a mixture of both their personal and professional lives, and this had a significant influence on how they framed messages, as well as when they posted. For others, participation in this study changed their perceptions about how social media can be used, and impacted significantly on their online identity.

Participants who had an established online identity tended to choose content based on how it integrated with their timeline, particularly if their online identity was a mix of personal and professional. One participant who has a Facebook profile with a large following connected to their professional identity explained that although they believed some of the more ‘hard-hitting’ content was likely to be effective, it was incompatible with their own Facebook feed, which was deliberately kept to a friendly and positive tone. “I like to keep my posts…kinda like Facebook-friendly to many family and friends back home.” (D1) That researcher also timed posts for this research project to fit appropriately with a range of other posts throughout the week, and developed a weekly ‘theme’ for their posts, which were posted on the same day each week.

### Considering potential reactions, likelihood of viewing

The potential sensitivity of messages for others was an important consideration for some participants. For example, there was a high level of awareness about when a message might ‘hit a bit too close to home’ for people dealing with a recent bereavement, especially for images that were particularly realistic. Certain times of the year also influenced choices; Christmas was considered a time when people may be thinking of absent family and friends, and therefore an important time to be sensitive to issues of grief and loss.

“…it was just too sad for me...it’s the harsh reality I know but I just didn’t feel comfortable posting it. So many people over the Christmas period were posting how much they missed their mums who have passed away and it was similar stories so that’s why I didn’t post cause I didn’t want to make people feel sad.” (A3)

People separated the importance and relevance of messages to their own lives from the expected likely reaction and interactions from their Facebook friends. In some cases, health information was already known by the participant, but they chose it because they thought it was probably not well known by people in their networks. This was also the case for some quit tips. On the other hand, sometimes the choice of post was deliberately chosen by the participant as a reminder to themselves in their own quitting journeys.

Several participants analysed the relevance of content to their own lives and contrasted it with those in their network, particularly in relation to age differences, smoking status and how long different people had been smoking. In some cases, posts were chosen by older participants that they did not think was relevant to them and others of their age, but might appeal to younger people and help stop them smoking. In other cases, the participant considered that content might be effective in particular age groups, but not for their networks:

“I didn’t share this for a couple of reasons…my friends and family don’t tend to view my quitting videos (unless I specifically tag them) and most of my friends and family is a different target group. I could not fault the video and as mentioned would be really good for young to mid age mothers and fathers groups where the guilt might hit them for their young family.”(D7, discussing week 2 option A)

### Change over time during project, impact on personal attitude

Several participants were nervous and apprehensive about posting content at the start of the study, especially smokers. Some were initially concerned about appearing to be hypocritical by posting messages about smoking, when many in their networks knew they were smokers. Both smokers and non-smokers also expressed concerns about potentially upsetting friends or feared they may be perceived as ‘preaching’, particularly in relation to disturbing or confronting images. Some participants believed that sharing the posts might be generating negative reactions which were not reflected in online interactions, with some suggesting that they had been ‘unfriended’ by people within their network..

“Some people have said, oh boring you’re not the same…..cause usually….I used to be putting up all the jokes…I think this [participation in this study] has changed me….I’ll put up a joke every now and then…but its more the serious side of things….Close to 10 people have unfriended me” (A1)

However, over time, some people became bolder and less concerned about their choices, particularly when they did not receive expected negative reactions. In these cases, there was a greater willingness to post messages that might be considered confronting and harsh as the study progressed. Several found that the reactions from their networks were either minimal or more positive than expected, and they became more willing to post confronting images. Consistent with this, some people started to post more than once per week, and also included personal messages to try and generate reactions. This was consistent with reports from friends and family within participants’ social networks in face-to-face interviews. Overall, friends and family reported that they perceived posts as being contextualised within the relationship they had with participants, generally as part of caring relationships. Often, they gave no visible indication of interacting with posts, even if the post was personally impactful (to be reported separately; manuscript in preparation).

The extent to which people were posting for their own purposes, rather than with the intention of influencing others, also changed over the course of the project. Particularly for participants who were trying to quit smoking, choices sometimes reflected a particularly critical time in their quit journey – for example, posting quit tips that were a reminder for themselves of practical steps they needed to take.

“After watching a video I noticed the cigarette tastes really funny. It does something and I don’t feel like smoking so much. Could be subconsciously…I’ve mentioned that to my [spouse]…” (A1, reflecting on videos viewed during the project)

For those participants who were finding it challenging to quit, the desire to post confronting images was often connected to their own perceived need for greater shock tactics.

“I picked the image I did because it was the most shocking and hardcore. I have to take this more serious. I’m traumatised by the image as I imagine many of my [Facebook friends and family] are. And I guess it’s one of my strategic moves to help me on my journey to quit. I need to. I didn’t choose the others because they weren’t as direct. I may change my mind and say it’s too close to home. My mum died of lung cancer at 66 years old. I am so scared. I need help.” (D6, discussing week 1 option C)

### Impact on phone credit/data usage

The potential impact on data usage and phone credit for people in their networks was a concern for some people, and sometimes for participants themselves. At times, this meant that longer videos were less likely to be shared, even if the content was considered good and interesting. One participant highlighted this as an issue that they considered when deciding whether to post a video about the (Australian government initiative) My Quit Buddy app:

“Although this is a beneficial video promoting the Quit Buddy app, that can be downloaded for free & has helped a lot of people I know of…. I’m thinking some of my family & friends have cheap phones and we love our music, videos, games and not to mention selfies, making no storage space on our phones and even with a memory card that space is used up. I’ve noticed this with a lot of young people in my family. Otherwise a great interactive app to have.” (A1, discussing week 22 option C)

## Discussion

This study has used community researchers to disseminate smoking prevention messages through their existing Facebook networks. The results show that posts which are child-focused, feature Indigenous content, and are perceived as practical, relevant and credible, with a direct and unambiguous message, are the most likely to be shared. Indirect, obscure, sarcastic and disgusting content without a clear message were less likely to be shared. Given that social media largely shifts the power to consumers to decide what content will be seen, this provides important lessons for planning social media-based smoking prevention strategies.

The popularity of child-focused messages might reflect the ambivalence many participants felt about at different stages of their participation in the study. Particularly for smokers, content focused on children represented ‘safe ground’ for posting, did not raise potential issues of hypocrisy, and reflects priorities that are common across most demographics, regardless of smoking status. In general, new information or a ‘fresh take’ on information was preferred; this was particularly true for some of the child-focused content. A good example was the popularity of the Thai Health advertisement; participants suggested that an Australian version of this ad featuring Indigenous people would be very well received, and likely to generate high levels of engagement. The study reinforces that international tobacco control content can be adapted for local messages on social media, as has been done over many years for mass media campaigns [41, 42].

The results raise questions about the potential effectiveness of using social media to disseminate confronting messages that arouse strong negative emotions, which have been proven to be successful in traditional media [33]. Although limited, previous research has found that mainstream Australian anti-smoking advertisements with strong graphic and emotive narratives are likely to be highly motivating for Indigenous smokers [43]. However, participants in this study wavered in their willingness for posting such messages. When they did share them, the personal relevance and content in the message needed to have additional motivating features beyond disgust. In interviews, they reported wanting content that was both practical and positive, which may reflect the fact that many of the study participants wanted to quit smoking. This contrasts with recent research among socially disadvantaged smokers which suggests that ‘why to’ quit messages are perceived as more effective than ‘how to’ messages [44]. However, although qualitative information suggested a strong preference for ‘how to’, much of the content selected by participants was ‘why to’.

An important finding is the reported strong personal impact of some messages on participants, which contrasts with the decision to not post these same impactful messages based on concern for potential sensitivities among their social media friends. This appears to be due to two separate processes. On the one hand, participants who were smokers appeared to be challenged about their own smoking, through the process of having to engage intensively with the material provided.

On the other hand, when deciding what messages to share, participants appeared to be guided by the fact that social media is used to build social capital, contribute to psychological well-being and construct a positive self-image [22]. In particular, having Facebook networks comprised of close social ties (including close offline relationships as in our study) is likely to influence the content people choose to share, as emotional support is an important component of such networks [23]. In this context of prioritising relationships, it makes sense that participants would choose messages that would not expose their friends to content which they found most uncomfortably challenging or which aroused strong negative emotions. These findings suggest that messages designed for sharing on social media need to be complementary to strong negative arousal messages that are spread through traditional broadcast media, point of sale warnings and graphic health warnings on tobacco packaging.

A surprising finding was the willingness to share posts about the impact of smoking on male fertility, given the gender-specific cultural sensitivity around reproductive health [45]. Most of the community researchers reported this focus being of interest because it was completely new information. This may be a case of deflection, as the majority of our participants were female and had either direct or indirect experience of smoking in pregnancy. Given the strong focus on smoking in pregnancy in tobacco control [46–48], particularly for Australian Indigenous women [49], there may be potential to include the impact of smoking on male fertility in messages, although care would need to be taken to consider appropriateness related to gender relations and specific socio-cultural and geographical contexts.

Similarly surprising was the lack of interest in posts that used environmental themes, as this was highlighted as an important Indigenous health issue in our first exploratory study for this project [36]. Even participants who consider the environment a high-priority issue only selected environmentally-focused messages when the issue was framed as concern for human health.

The concerns about data and credit usage are significant for planning social media-based health promotion campaigns, particularly in communities where most access to social media relies on expensive mobile phone data. It should be noted that in this study, we did not have the ability for participants to embed videos in Facebook stream for automatic play, which minimises the impact on data. Being able to do so may make it likely for some videos to be more widely shared. However, given the risk of people scrolling past content even if video plays automatically, care should be taken to ensure the first frames and/or thumbnails of a video are engaging and attention-grabbing. Our results show that the thumbnail and title can be a deciding factor in engaging with or sharing content.

An important insight from this study was the extent to which people’s attitudes to posting content changed over time. Despite initial reticence, both smokers and non-smokers showed an increasing willingness to post a range of content, particularly as fears about anticipated negative reactions proved to be unfounded. This is consistent with insights from the neuroscience of social media, which shows that posting involves a self-referential cognition network loop consisting of thinking about oneself, sharing experiences and opinions, and receiving feedback which then prompts further self-appraisal. Posts that receive even minimalistic positive cues such as likes, further activate the sense of reward, which in turn provides further incentive to post [24]. This process may explain participants’ confidence increasing during the course of the study, along with personal investment and engagement in the study.

The use of paid social media ‘influencers’ is a strategy used by the tobacco industry in a range of contexts [50]; our results show that tobacco control social media ‘champions’ and ‘influencers’, can be nurtured. This approach has several benefits: in addition to boosting active community engagement and disseminating messages through targeted networks, it is relatively low cost, while also providing casual employment opportunities to local community members. Tobacco control messages were a small proportion of health messages observed in the first exploratory study for this project [36]; this study shows that it is possible to cultivate a network of local community ‘influencers’ to disseminate tobacco control messages through personal social media networks.

### Strengths and limitations

The strengths of this study include the detailed contextual information about decision-making for sharing tobacco control content within real-world networks, and the diversity of participants in terms of age range, smoking status, socioeconomic status, and initial attitudes to the study. A limitation of the study is that participants were from two small regional cities, which may limit generalisability to other services and geographical areas. Furthermore, the fact that participants were paid limits the applicability to the study findings to generating ‘organic’ sharing of messages on Facebook.

This study did include examination, not reported here, of how the participants’ Facebook friends reacted to posts, both through visible online interactions and non-visible offline responses. These results will be reported in a separate paper (manuscript in preparation).

## Conclusions

The results from this study suggest that anti-smoking content designed to be shared on social media should complement, rather than attempt to replicate, messages from tobacco control mass media campaigns. In particular, content should take into account the role of social media in strengthening relationships and developing social capital, both of which are likely to be prioritised above posting messages which are confronting and designed to arouse strong negative emotions. Participants in this study needed to perceive content as likely to be helpful to their personal networks, preferably because it contained new and/or supportive information. Tobacco control social media messages that include a focus on children, feature culturally-specific and locally tailored content, are practical, relevant, credible, and have direct and unambiguous messages were the most likely to be shared. Indirect, obscure, sarcastic and disgusting content without a clear message are less likely to be shared. This study shows the potential for health services to incorporate a strategy of using paid local social media ‘champions’ or ‘ambassadors’ to disseminate tobacco control messages on Facebook through community networks.
